# REV-ERBα influences the stability and nuclear localization of the glucocorticoid receptor

**DOI:** 10.1242/jcs.190959

**Published:** 2016-11-01

**Authors:** Takashi Okabe, Rohit Chavan, Sara S. Fonseca Costa, Andrea Brenna, Jürgen A. Ripperger, Urs Albrecht

**Affiliations:** Dept. of Biology, Biochemistry, University of Fribourg, Fribourg 1700, Switzerland

**Keywords:** Nuclear receptor, Circadian clock, Glucocorticoid receptor, REV-ERBα, HSP90

## Abstract

REV-ERBα (encoded by *Nr1d1*) is a nuclear receptor that is part of the circadian clock mechanism and regulates metabolism and inflammatory processes. The glucocorticoid receptor (GR, encoded by *Nr3c1*) influences similar processes, but is not part of the circadian clock, although glucocorticoid signaling affects resetting of the circadian clock in peripheral tissues. Because of their similar impact on physiological processes, we studied the interplay between these two nuclear receptors. We found that REV-ERBα binds to the C-terminal portion and GR to the N-terminal portion of HSP90α and HSP90β, a chaperone responsible for the activation of proteins to ensure survival of a cell. The presence of REV-ERBα influences the stability and nuclear localization of GR by an unknown mechanism, thereby affecting expression of GR target genes, such as IκBα (*Nfkbia*) and alcohol dehydrogenase 1 (*Adh1*). Our findings highlight an important interplay between two nuclear receptors that influence the transcriptional potential of each other. This indicates that the transcriptional landscape is strongly dependent on dynamic processes at the protein level.

## Abstract (precis)

**Summary:** Nuclear localization of the glucocorticoid receptor that is dependent on the time of day is altered at the post-translational level by the absence of REV-ERBα, affecting expression GR target genes.

## INTRODUCTION

In mammals, the circadian clock system regulates many physiological, biochemical and behavioral processes with the suprachiasmatic nuclei (SCN) as the main coordinating entity to synchronize all cellular clocks in the body (Panda et al., 2002; Reppert and Weaver, 2002). At the cellular level, the circadian clock mechanism is controlled by genetically determined networks of transcription–translation feedback loops involving clock genes, including period (*Per*, for which there are three genes *Per1–Per3*), cryptochrome 1 and 2 (*Cry1* and *Cry2*), *Bmal1* (also known as *Arntl*) and *Clock* (Reppert and Weaver, 2001). The transcription factors CLOCK and BMAL1 heterodimerize and activate the expression of *Per* and *Cry* genes by binding to E-box elements in their promoters. CRY and PER proteins form oligomers that are transported from the cytoplasm to the nucleus, where they repress their own transcription by inhibiting BMAL1–CLOCK activity. BMAL1–CLOCK also induces the expression of the nuclear receptor REV-ERBα (also known as NR1D1), which represses the transcription of *Bmal1* through direct binding to a REV-ERBα response element (RORE) within the *Bmal1* promoter (Buhr and Takahashi, 2013). *Rev-erbα* (*Nr1d1*) is expressed in a variety of tissue types, including brown fat, skeletal muscle and liver (Woldt et al., 2013; Everett and Lazar, 2014). In addition to its action in the circadian clock mechanism, REV-ERBα has been implicated in adipogenesis (Kumar et al., 2010; Laitinen et al., 2015), muscle differentiation (Downes et al., 1995), liver metabolism (Bugge et al., 2012; Cho et al., 2012) and neurogenesis (Schnell et al., 2014).

The glucocorticoid receptor (GR, encoded by *Nr3c1*) belongs to the superfamily of steroid, thyroid and retinoid acid receptor proteins that function as ligand-dependent transcription factors (Evans, 1988). A common feature of GR is an obligate interaction with heat-shock protein 90 (HSP90α and HSP90β, hereafter HSP90) before hormone-dependent activation (Kirschke et al., 2014) and this interaction is also known to be important in regulating GR stability (Siriani et al., 2005; Sultana et al., 2013). Upon binding its cognate ligand (glucocorticoids), GR undergoes conformational changes, dissociates from HSP90 binding, and subsequently translocates to the nucleus and binds to conserved palindromic DNA sequences, the glucocorticoid responsive element (GRE) (Berg, 1989). Depending on the target gene, hormone-activated GR can stimulate or inhibit gene transcription and direct binding of activated GR on GRE is the main mechanism of regulation associated with glucocorticoid-mediated transactivation (De Bosscher et al., 2003). For example, GR exerts anti-inflammatory action in part by antagonizing nuclear factor κB (NF-κB), which is known to activate genes coding for pro-inflammatory cytokines such as tumor necrosis factor (TNF) (De Bosscher et al., 2000) through the upregulation of the expression of the NF-κB inhibitor IκB proteins (Scheinman et al., 1995; Almawi and Melemedjian, 2002). Recent studies have also indicated that GR can bind as a monomer or homodimer to target genes, depending on absence or presence of glucocorticoids (Lim et al., 2015).

A role of REV-ERBα in inflammation has been reported (Gibbs et al., 2012, 2014), and REV-ERBα appears to regulate NF-κB-responsive genes in human vascular smooth muscle cells (Migita et al., 2004). Overexpression of REV-ERBα upregulates inflammatory markers, like IL-6 and COX-2, as well as nuclear translocation of NF-κB (Migita et al., 2004), suggesting that REV-ERBα might play an important role in the regulation of NF-κB responsive genes. Among NF-κB controlled genes, TNF plays a crucial role in alcohol liver diseases (Beier and McClain, 2010; Gao et al., 2011). Ethanol increases NF-κB activation through reactive oxygen species (ROS)-dependent pathways (Wang et al., 2015) and production of TNF in hepatocytes is controlled by the transcriptional activation of NF-κB through the degradation of IκBα (Fan et al., 2004).

Given that REV-ERBα might be involved in regulation of NF-κB and that GR exerts anti-inflammatory functions through the upregulation of the expression of IκBα, the correlation between REV-ERBα and GR is of particular interest. Therefore, we studied the relationship between REV-ERBα and GR. Here, we show that REV-ERBα and GR bind to HSP90 at different sites, affecting their stability and subcellular localization by unknown mechanisms. As a consequence the regulation of target genes is affected.

## RESULTS

### REV-ERBα influences the GR at the protein but not at the transcript level

**Fig. 1.**
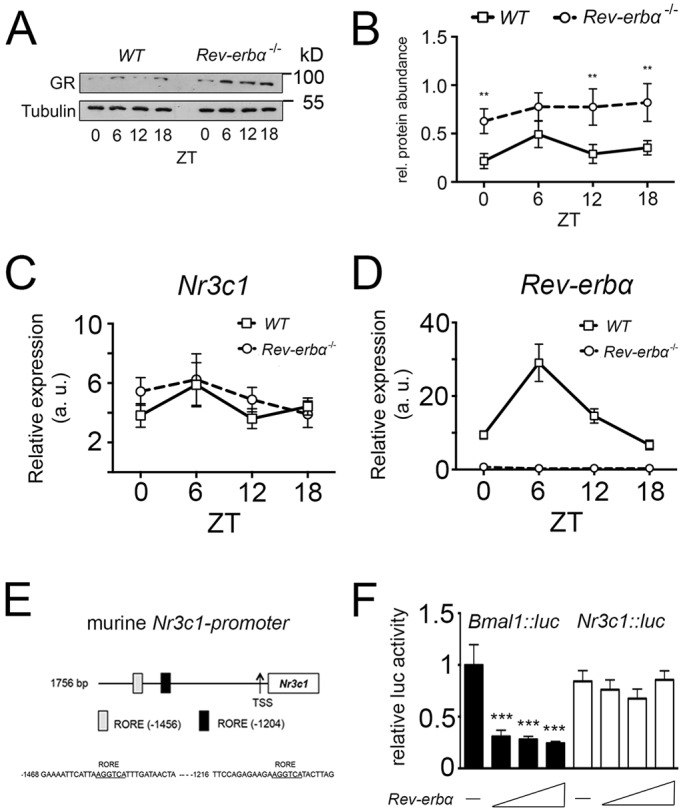
**GR protein, but not mRNA, is highly expressed in *Rev-Erbα^−/−^* mice.** (A) Western blots of liver extracts from wild-type (WT) and *Rev-erbα*^−/−^ mice showing expression of GR and tubulin using their respective antibodies. Liver whole-cell lysates were extracted from wild-type and *Rev-erbα*^−/−^ mice every 6 h, beginning at Zeitgeber time (ZT) 0 for one 24-h cycle. (B) Quantification of the GR signal normalized to tubulin (mean±s.d., *n*=3). **P<0.01 (two-way ANOVA). (C) Quantification of glucocorticoid receptor (*Nr3c1*) mRNA in the liver. Liver total RNA was extracted from wild-type and *Rev-erbα*^−/−^ mice every 6 h, beginning at ZT0 for one 24-h cycle, and *Nr3c1* transcripts were quantified (mean±s.d., *n*=3). (D) Quantification of *Rev-erbα* (*Nr1d1*) mRNA in the liver. Liver total RNA was extracted from wild-type and *Rev-erbα*^−/−^ mice every 6 h, beginning at ZT0 for one 24-h cycle, and *Rev-erbα* transcripts were quantified (mean±s.d., *n*=3). (E) Schematic representation of murine *Nr3c1* promoter with its two REV-ERBα response elements (ROREs). (F) Luciferase assay of *Nr3c1::luc* reporter constructs with *Rev-erbα* overexpression plasmids in NIH3T3 cells. *Bmal1::luc* was used as a positive control (mean±s.d., *n*=3). ***P<0.001 (one-way ANOVA with Bonferroni post-test).

In order to test the possibility of whether GR and REV-ERBα influence each other, we tested the expression of GR in *Rev-erbα^−/−^* animals and compared it with the expression in wild-type (WT) animals using liver extracts. Western blotting revealed that GR protein was expressed in an almost constant manner over 1 day in the liver and that this expression was strongly increased in *Rev-erbα^−/−^* livers (Fig. 1A). Quantification of several experiments indicated that this increase was statistically significant at the time points zeitgebertime (ZT) 0, 12 and 18, where ZT0 is lights on and ZT12 is lights off (Fig. 1B). This suggests that *Rev-erbα* affects GR protein accumulation.

As a next step, we tested the possibility that REV-ERBα regulates the expression of GR (*Nr3c1*) at the transcriptional level. Therefore, we extracted mRNA from livers of wild-type and *Rev-erbα^−/−^* mice. Quantitative real-time PCR (qRT-PCR) revealed that the level of *Nr3c1* mRNA was similar in wild-type and *Rev-erbα^−/−^* livers (Fig. 1C), whereas *Rev-erbα* mRNA was absent in the *Rev-erbα^−/−^* animals (Fig. 1D). Given that the murine *Nr3c1* promoter contains REV-ERBα-binding sites (ROREs, Fig. 1E), we tested whether this promoter cloned in front of a luciferase reporter gene (*Nr3c1::luc*) was repressed by REV-ERBα in a transfection experiment using NIH3T3 cells. We observed that increasing concentrations of *Rev-erbα* expression vector did not lead to a reduction of luciferase activity when using the *Nr3c1::luc* reporter in contrast to the positive control *Bmal1::luc* reporter (Fig. 1F). This result indicates that the *Nr3c1* promoter is not directly regulated by the repressor activity of REV-ERBα, which is consistent with the unaltered levels of *Nr3c1* mRNA in *Rev-erbα^−/−^* mice (Fig. 1C). Therefore, the increase in GR protein in the liver of *Rev-erbα^−/−^* animals probably occurs post-transcriptionally and/or post-translationally.

### GR and REV-ERBα both bind to HSP90 affecting GR stability

**Fig. 2.**
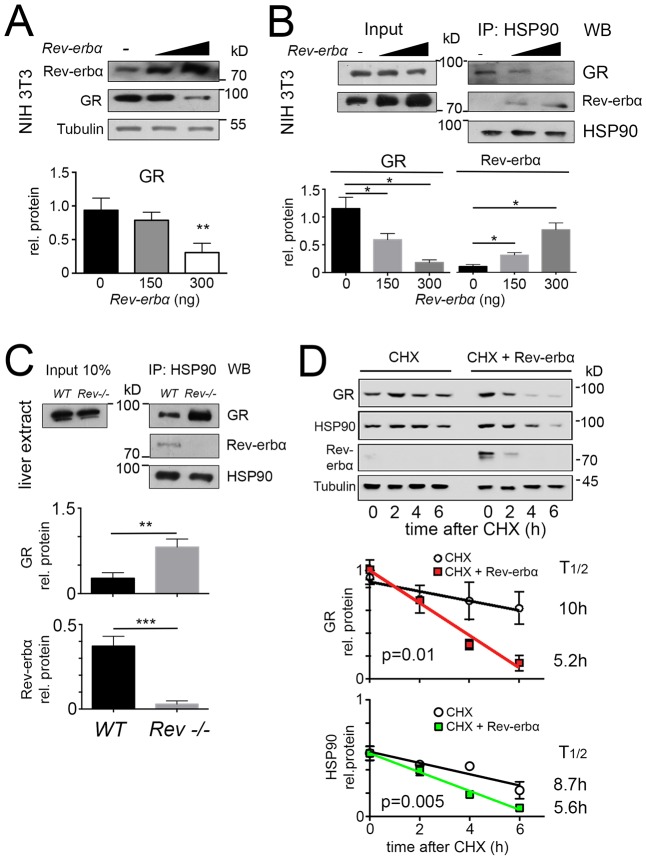
**REV-ERBα and GR compete with each other for binding to HSP90, resulting in the destabilization of GR protein.** (A) Western blot of whole-cell extracts from NIH3T3 cells transfected with increasing amounts of expression vector for *Rev-erbα* with indicated antibodies. The lower panel shows the quantification of GR signal (mean±s.d., *n*=3). **P<0.01 (*t*-test). (B) Western blot (WB) of HSP90 immunoprecipitates (IP: HSP90) and whole-cell extracts (Input) from NIH3T3 cells transfected with the expression vector for *Rev-erbα*. The lower panel shows the quantification of the GR and REV-ERBα signal in HSP90 immunoprecipitates normalized to that of HSP90 (mean±s.d., *n*=3). *P<0.05 (one-way ANOVA with Bonferroni post-test). (C) Western blot of HSP90 immunoprecipitates (IP: HSP90) and whole-cell extracts (Input) from liver of wild-type (WT) and *Rev-erbα*^−/−^ mice (*Rev−/−*). The lower panel shows the quantification of the GR and REV-ERBα signal in HSP90 immunoprecipitates normalized to that of HSP90 (mean±s.d., *n*=3). **P<0.01, ***P<0.001 (*t*-test). (D) Western blot of whole-cell extracts from NIH3T3 cells transfected with expression vector for *Rev-erbα* and treated with cycloheximide (CHX; 100 μg/ml). The middle panel shows the quantification of GR signal. GR half-life with CHX alone is 10 h (R^2^=0.84, *n*=4) and with CHX and *Rev-erbα* it is 5.2 h (R^2^=0.98, *n*=4). The lower panel shows the quantification of HSP90 signal. HSP90 half-life with CHX alone is 8.7 h (R^2^=0.87, *n*=4) and with CHX and *Rev-erbα* it is 5.6 h (R^2^=0.99, *n*=4). Results are mean±s.d. The *P*-values given on the figure were calculated with a two-way ANOVA test.

Lack of the *Rev-erbα* gene leads to increased GR protein in the liver (Fig. 1A). If REV-ERBα affected GR levels post-transcriptionally, overexpression of *Rev-erbα* would lead to a reduction of GR protein. In order to test this hypothesis, we overexpressed differing amounts of REV-ERBα in NIH 3T3 cells. We observed that increasing amounts of REV-ERBα protein lead to a reduction of GR protein in these cells as revealed by western blotting (Fig. 2A). One explanation for this might be that GR is less stable in presence of excess REV-ERBα. Because HSP90 interacts with GR and thereby stabilizes it (Siriani et al., 2005; Sultana et al., 2013), we hypothesized that REV-ERBα might also bind to HSP90 and modulate GR stability. Therefore, we immunoprecipitated HSP90 in presence of increasing amounts of REV-ERBα, and tested the amounts of REV-ERBα and GR, respectively, that were co-precipitated with HSP90 by western blotting (Fig. 2B). Interestingly, increasing amounts of REV-ERBα reduced the amount of GR that was co-precipitated with HSP90 and increased the amount of REV-ERBα that was co-precipitated with HSP90 in a dose-dependent manner (Fig. 2B). Consistent with these observations, we found that the amount of HSP90 immunoprecipitated GR was increased in liver extracts from *Rev-erbα^−/−^* compared to wild-type animals (Fig. 2C). Therefore, it is very likely that REV-ERBα modulates GR levels by binding to HSP90. In order to test whether REV-ERBα binding to HSP90 had an influence on the stability of GR, we treated NIH 3T3 cells with cycloheximide, a translation inhibitor, in presence of endogenous REV-ERBα (Fig. 2D, top, left side) and in presence of a plasmid leading to overexpression of REV-ERBα (Fig. 2D, top, right side). We found that the half-life of GR protein was reduced by overexpression of REV-ERBα and decreased from 10 h to 5.2 h (Fig. 2D, middle panel), indicating that overexpression of REV-ERBα affects the half-life of GR protein. Likewise, REV-ERBα overexpression affected the half-life of HSP90, reducing it from 8.7 h to 5.6 h (Fig. 2D, bottom panel). Hence, protein stability of GR appears to be related to HSP90 levels, which are directly or indirectly affected by REV-ERBα. We cannot exclude, however, the possibility that indirect effects stemming from changes in HSP90 levels can affect GR function through other HSP90 client proteins.

### REV-ERBα affects diurnal subcellular localization of GR

**Fig. 3.**
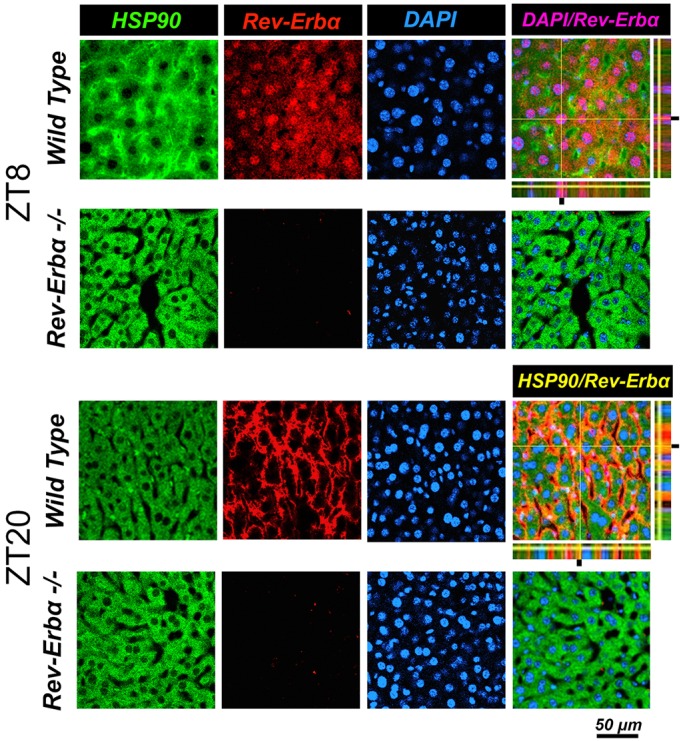
**Cellular distribution of REV-ERBα at different times of day.** Immunohistochemistry in the liver of wild-type and *Rev-Erbα*^−/−^ mice at ZT8 and ZT20. Staining with antibodies recognizing HSP90 (a marker for cytoplasm) and REV-ERBα are in green and red, respectively. Nuclei are stained with DAPI (blue). The pink color indicates nuclear REV-ERBα and the yellow color cytoplasmic REV-ERBα. Note the reversed cellular distribution of REV-ERBα compared to GR (Fig. 4) in wild type. Scale bar: 50 µm.

**Fig. 4.**
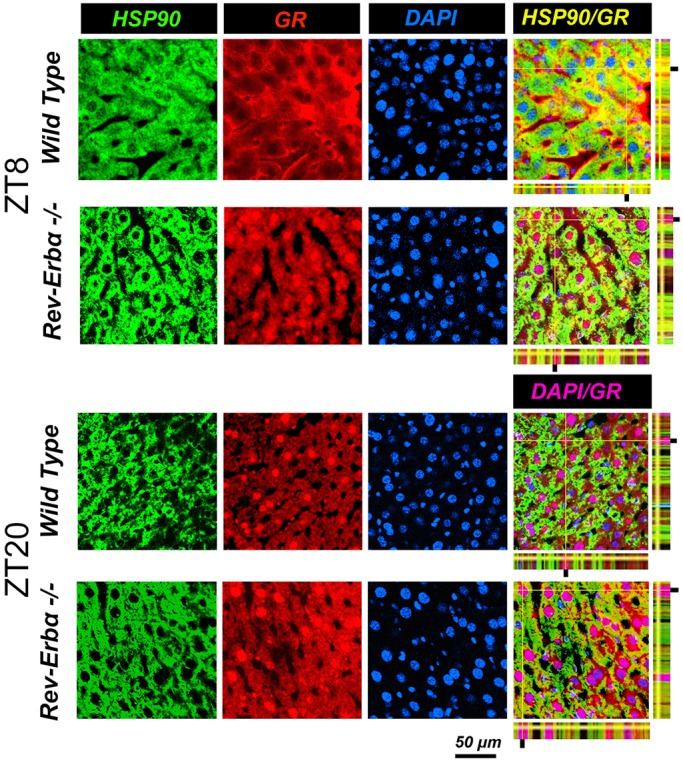
**The change in cellular distribution of GR that is dependent on the time of day is abolished in *Rev-erbα*^−/−^ mice.** Immunohistochemistry in the liver of wild-type and *Rev-Erbα*^−/−^ mice at ZT8 and ZT20. Staining with antibodies recognizing HSP90 (marker for cytoplasm) and GR are in green and red, respectively. Nuclei are stained with DAPI (blue). The pink color indicates nuclear GR and the yellow color cytoplasmic GR. Scale bar: 50 µm.

**Fig. 5.**
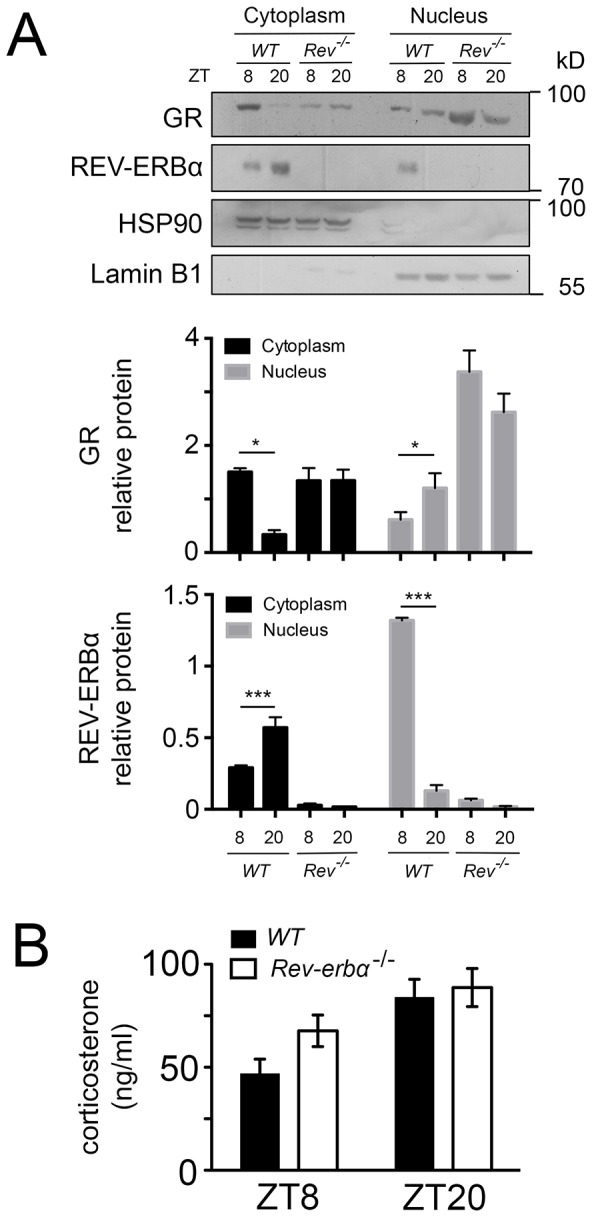
**REV-ERBα and GR reciprocally influence their nuclear localization.** (A) Western blot of the cytoplasm and nuclear fraction of the liver of wild-type (WT) and *Rev-Erbα*^−/−^ (*Rev*^−/−^) mice at ZT8 and ZT20. The lower panel shows the quantification of signals (mean±s.d., *n*=4). *P<0.05, ***P<0.001 (one-way ANOVA with Bonferroni post-test). (B) The levels of corticosterone in the plasma of wild-type (black bars) and *Rev-Erbα*^−/−^ (white bars) mice revealed by ELISA (*n*=6, mean±s.d.).

In a next step, we wanted to know how the absence of REV-ERBα affects nuclear and cytoplasmic localization of GR in liver cells. Immunohistochemistry on liver tissue from wild-type and *Rev-erbα^−/−^* mice harvested at ZT8 and ZT20 revealed profound alterations in the nuclear localization of GR that was dependent on both the presence of REV-ERBα and the time of day (Figs 3 and 4). At ZT8 REV-ERBα was strongly expressed in the nucleus of wild-type liver and absent in *Rev-erbα^−/−^* liver whereas HSP90 was expressed in both genotypes in the cytoplasm at both time points ZT8 and ZT20 (Fig. 3). At ZT20 REV-ERBα was mainly in the cytoplasm in wild-type (at reduced levels as compared to nuclear REV-ERBα at ZT8 as determined by the quantification, see below Fig. 5A) and absent in liver of *Rev-erbα^−/−^* mice (Fig. 3), illustrating the diurnal pattern of REV-ERBα subcellular localization. Interestingly, this pattern was inverted for GR in wild-type animals (Fig. 4). GR was mainly cytoplasmic at ZT8 and nuclear at ZT20. However, in *Rev-erbα^−/−^* mice, this diurnal pattern of subcellular localization of GR was absent and GR was present at both time points in the nucleus as well as in the cytoplasm (Fig. 4). In order to quantify these observations, we performed a cytoplasmic and a nuclear fractionation. REV-ERBα was mainly nuclear at ZT8, whereas GR was mainly cytoplasmic at this time point in wild-type mice (Fig. 5A). The inverse was observed at ZT20 when REV-ERBα was cytoplasmic and GR was nuclear. In *Rev-erbα^−/−^* mice, GR was increased, especially in the nucleus, and the temporal variations in both the nucleus and cytoplasm were absent (Fig. 5A), which is consistent with the immunohistochemistry experiments (Figs 3 and 4). These REV-ERBα-associated changes in nuclear localization of GR were most likely not mediated by corticosterone, because its levels were not significantly different between wild-type and *Rev-erbα^−/−^* animals at ZT8 and ZT20 (Fig. 5B). Taken together, these observations indicate that the presence of REV-ERBα inversely correlates with a diurnal pattern in the subcellular distribution of GR.

### Diurnal interaction of GR and REV-ERBα with HSP90 in liver and sites of interaction on HSP90

**Fig. 6.**
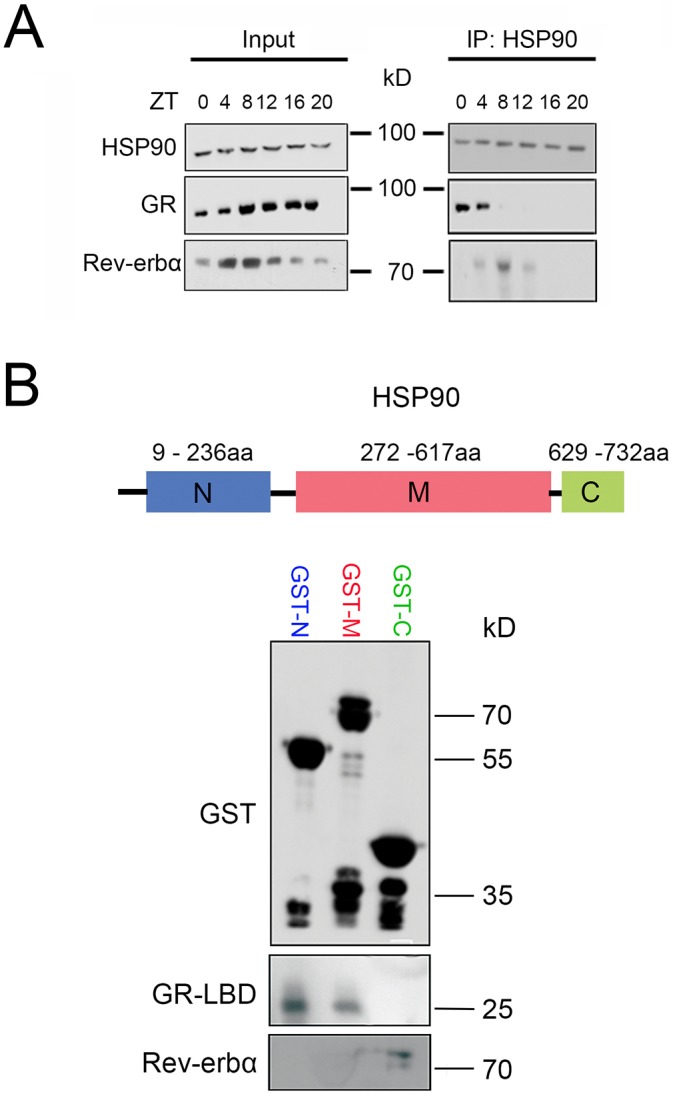
**Diurnal interaction of GR and REV-ERBα with HSP90 in liver and sites of interaction on HSP90.** (A) Western blot of HSP90 immunoprecipitates (IP: HSP90) (right panel) and whole-cell extracts (Input) from liver cells of wild-type mice. Livers were collected at ZT0, 4, 8, 12, 16 and 20. (B) Pulldown assays with an N-terminal (N, blue), middle (M, red) and C-terminal (C, green) HSP90 fragment fused to GST that had been expressed in bacteria by IPTG induction and purified with gluthatione sepharose resin. The complex-resin–GST proteins were coimmunoprecipitated with either LBD-GR or REV-ERBα, both fused to a His tag, that had been also expressed in bacteria, purified using nickel resin and eluted using imidazole. The pulldown was detected by western blotting. Anti-GST antibodies reveal a 55-kDa GST-N protein, a 70-kDa GST-M protein and a 40-kDa GST-C protein. An anti-His tag antibody reveals a 25-kDa GR-LBD and 75-kDa REV-ERBα protein in the GST-N, GST-M and GST-C fractions, respectively.

In order to determine the timing of GR and REV-ERBα interaction with HSP90 in the liver, we collected total protein extracts from liver tissue around the clock from mice kept in a 12-h-light–12-h-dark cycle. Western blotting revealed constant levels of HSP90 and GR proteins in extracts from livers of wild-type mice (Fig. 6A, input). REV-ERBα levels were diurnal with a peak around ZT4–ZT8 (Fig. 6A, input). Immunoprecipitation of HSP90 co-precipitated GR at ZT0 and ZT4 (Fig. 6A, right panel). REV-ERBα was co-precipitated at ZT4, ZT8 and ZT12 with a peak at ZT8. These data indicated a sequential interaction of GR and REV-ERBα with HSP90 with an overlap at ZT4.

Because the interaction of the two nuclear receptors with HSP90 was sequential, we aimed to elucidate whether GR and REV-ERBα bind to the same or different sites on the HSP90 protein. Three different fragments of HSP90 (Fig. 6B, top scheme) fused at the N-terminus to GST were used to determine interaction with the ligand-binding domain (LBD) of GR and full-length REV-ERBα. Three HSP90 constructs were overexpressed in bacteria and the resulting proteins showed a size of about 55 kDa for GST-N, 70 kDa for GST-M and 40 kDa for GST-C. A pulldown assay revealed interaction of the ligand-binding domain of GR (GR-LBD) with GST-N and GST-M, whereas REV-ERBα interacted only with GST-C (Fig. 6B). These results indicate that GR and REV-ERBα interact at different sites with HSP90 and most likely do not compete for the same site.

### Expression of GR target genes are affected by REV-ERBα and vice versa

**Fig. 7.**
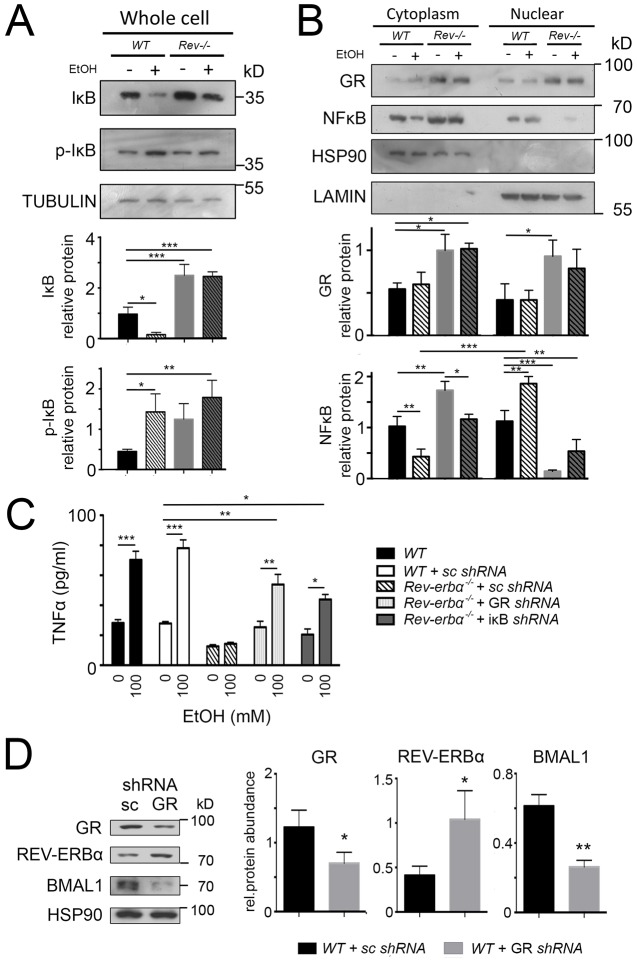
**REV-ERBα and GR affect target genes of each other in primary cultured hepatocytes.** Western blots of primary cultured hepatocytes of wild-type (WT) and *Rev-Erbα*^−/−^ (*Rev*^−/−^) mice. Cells were incubated with dexamethasone (1 µM) for 24 h, and culture medium was changed for one with or without 100 mM ethanol. (A) IκBα (IκB) and p-IκBα (p-IκB) specific antibodies reveal an increase in expression of IκBα in *Rev-Erbα*^−/−^ cells and an increase of p-IκBα in wild type after ethanol treatment (mean±s.d., *n*=3). *P<0.05, **P<0.01, ***P<0.001 (one-way ANOVA with Bonferroni post-test). (B) GR- and NF-κB-specific antibodies reveal increased expression of GR in *Rev-Erbα*^−/−^ hepatocytes, increased nuclear NF-κB signal in wild type cells and a decreased signal of NF-κB in *Rev-Erbα*^−/−^ cells after ethanol treatment (mean±s.d., *n*=3). *P<0.05, **P<0.01, ***P<0.001 (one-way ANOVA with Bonferroni post-test). (C) ELISA of TNF in primary cultured hepatocytes from wild-type and *Rev-Erbα*^−/−^ mice. Cells were treated with GR or IκBα shRNA lentiviral particles. Scrambled shRNA lentiviral particles (sc) were used as a negative control. At 24 h after transduction, dexamethasone (1 µM) was added and cells were incubated for 24 h, and then the culture medium was changed for one with or without 100 mM ethanol (mean±s.d., *n*=3). *P<0.05, **P<0.01, ***P<0.001 (one-way ANOVA with Bonferroni post-test). (D) Western blot of primary cultured hepatocytes of wild-type mice. Cells were treated with GR shRNA lentiviral particles. Scrambled shRNA lentiviral particles were used as a negative control. The right panel shows the quantification of signals (mean±s.d., *n*=3). *P<0.05, **P<0.01 (*t*-test).

In order to evaluate the functional consequences of the interplay between REV-ERBα and GR, we studied target genes of these two transcription factors in dexamethasone-treated hepatocytes. GR exerts anti-inflammatory action in part by antagonizing NF-κB, which is known to activate genes coding for pro-inflammatory cytokines, such as TNF (De Bosscher et al., 2000), through the upregulation of the expression of IκBα (Scheinman et al., 1995; Almawi and Melemedjian, 2002), a direct GR target (Fig. S1). To examine the effects of REV-ERBα on the NF-κB signaling pathway, we isolated primary hepatocytes at ZT8 and applied ethanol to these cells (Fig. 7), which is known to induce inflammatory reactions. In wild-type hepatocytes ethanol treatment led to an increase of phosphorylated IκBα (p-IκBα) and a reduction of unphosphorylated IκBα (Fig. 7A). As a consequence NF-κB translocated from the cytoplasm into the nucleus (Fig. 7B). In contrast, unphosphorylated IκBα accumulated in *Rev-erbα^−/−^* hepatocytes, probably as a consequence of increased GR levels in these animals (Fig. 7A,B), leading to reduced amounts of NF-κB in the nucleus. To test the above observations, we measured the levels of TNF secretion. Upon ethanol treatment TNF levels increased even in the presence of scrambled short hairpin RNA (shRNA) molecules (Fig. 7C). In contrast, ethanol did not induce TNF secretion in hepatocytes from *Rev-erbα^−/−^* animals. However, knocking-down GR or IκBα rescued, at least in part, ethanol-induced TNF secretion of *Rev-erbα^−/−^* hepatocytes (Fig. 7C). This indicates that REV-ERBα influences ethanol-induced TNF secretion through GR and IκBα in hepatocytes. For this process, however, glucocorticoids, GR ligands, are essential, because glucocorticoid-depleted *Rev-erbα^−/−^* hepatocytes display similar levels of p-IκBα, IκBα and NF-κB compared to wild-type heptaocytes (Fig. S2A; Fig. 2B). Therefore, TNF can be normally induced in glucocorticoid-depleted *Rev-erbα^−/−^* hepatocytes upon ethanol treatment (Fig. S2C).

Apparently, REV-ERBα affects GR targets, but does GR also affect REV-ERBα targets? We isolated wild-type hepatocytes and cultured them in presence of scrambled shRNA or GR shRNA. We observed that GR shRNA knocked down GR at the mRNA (Fig. S3) and the protein levels (Fig. 7D), which led to an increase in REV-ERBα accompanied by a reduction in expression of its target BMAL1 (Fig. S3; Fig. 7D). This is consistent with previous findings (Preitner et al., 2002). Hence, GR can also affect the expression of at least some REV-ERBα targets.

### REV-ERBα and GR modulate the diurnal metabolic landscape

REV-ERBα has been strongly implicated in the regulation of metabolism (Bugge et al., 2012; Cho et al., 2012). In order to test whether metabolic changes observed in livers of *Rev-erbα^−/−^* mice are related to GR, we compared genes changed in *Rev-erbα^−/−^* animals with genes regulated by GR (Phuc Le et al., 2005). If the hypothesis that REV-ERBα and GR influence expression of each other's target genes were correct, we would expect to be able to identify changes in expression of direct GR targets in *Rev-erbα^−/−^* animals.

**Fig. 8.**
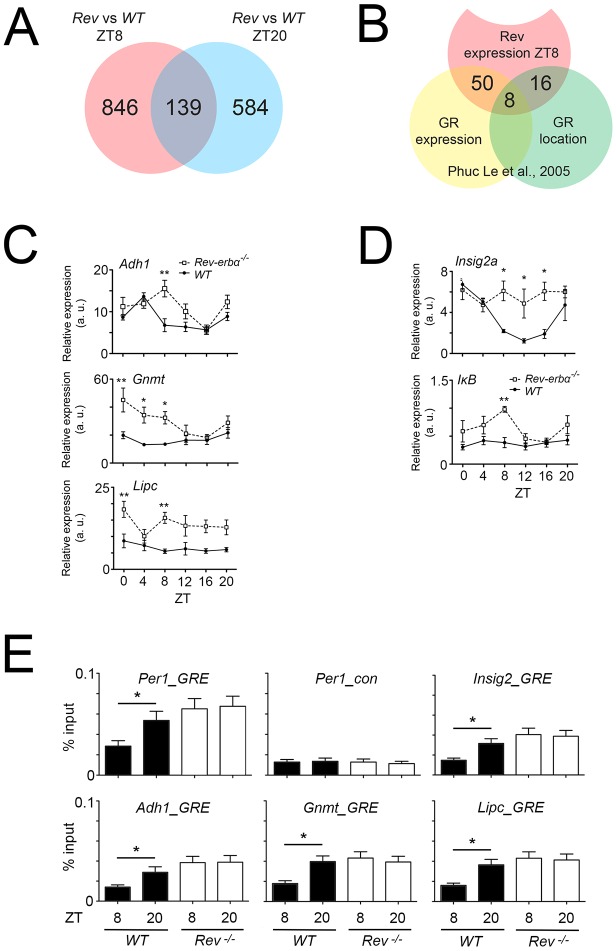
**Gene expression changes in *Rev-erbα*^−/−^ liver and comparison with GR target genes.** (A) Venn-diagram based on the RNA sequencing analysis of *Rev-erbα*^−/−^ and control (wild-type, WT) livers at ZT8 (red) and ZT20 (blue). (B) Comparison of the 846 genes altered at ZT8 (red) in *Rev-Erbα*^−/−^ liver with datasets from Phuc Le et al. (2005) (yellow and green, ZT4–ZT8). The yellow circle represents gene expression alterations in the liver after dexamethasone treatment assessed by microarray. The green circle represents genes detected by a ChIP-on-chip assay using an antiserum raised against GR to reveal chromatin bound by GR. (C) Verification of three of the eight genes in common to all three evaluations: alcohol dehydrogenase 1 (*Adh1*), glycine N-methyltransferase (*Gnmt*), and hepatic lipase C (*Lipc*). Values are mean±s.e.m., *n*=3. *P<0.05, **P<0.01 (two-way ANOVA and Tukey's post-test). (D) Verification of the bona fide REV-ERBα target gene *Insig2a* and the GR target gene *IκBα* (*Nfkbia*) (values are mean±s.e.m., *n*=3. *P<0.05, **P<0.01 (two-way ANOVA and Tukey's post-test). (E) Chromatin immunoprecipitation assay revealing the time-dependent interaction of GR with the promoters of *Per1*, *Insig2*, *Adh1*, *Gnmt* and *Lipc*. Values are mean±s.e.m., *n*=8, *P<0.05 (one-way ANOVA with Bonferroni-post test).

In a first step, we performed RNA sequencing of wild-type and *Rev-erbα^−/−^* livers at ZT8 and ZT20 and compared the data in order to identify transcripts that are altered at ZT8 but not at ZT20 (Fig. 8A). Because REV-ERBα is highly expressed at this time point, the changes in REV-ERBα are substantial between wild-type and *Rev-erbα^−/−^* animals, and, hence, the changes in GR nuclear localization would also be substantial. We identified 846 genes altered in expression in *Rev-erbα^−/−^* livers (Fig. 8A). We further analyzed up- or down-regulated genes between *Rev-erbα^−/−^* and wild-type liver at ZT8 with a threshold of 1.5 or more. The resulting 25 genes were submitted to ontology selection, and ordered according to biological process or molecular function. We identified two genes involved in immune system processes and 13 genes involved in metabolic processes, including *Bmal1*, a clock gene directly regulated by REV-ERBα (Preitner et al., 2002).

Then, we compared the 846 differentially expressed genes between *Rev-erbα^−/−^* and wild-type at ZT8 (Fig. 8A) with genes regulated by GR at around ZT4–ZT8 (Phuc Le et al., 2005). From these 846 genes, we found 50 genes in common with a list of genes altered in expression in livers after dexamethasone injection (Fig. 8B, overlap yellow and red circles; Table S1, green marked genes). Interestingly this list contained two genes involved in metabolic processes that were more than 1.5 times upregulated in *Rev-erbα^−/−^* liver (*Cdkn1a* and *Ddc*). From the 846 genes, 16 genes were also present in a list of genes derived from a previous ChIP-on-Chip experiment at around ZT4–ZT8 (Phuc Le et al., 2005) using anti-GR antibodies, representing differences in promoter-binding after dexamethasone injection into livers (Fig. 8B, overlap red and green circles; Table S1, blue marked genes). In common between the expression analysis and the location analysis were eight genes that most likely represent direct GR targets altered in *Rev-erbα^−/−^* livers (Fig. 8B, overlap yellow, red and green circles; Table S1, yellow marked genes).

From these eight potential GR target genes, we verified three genes that displayed large changes by qRT-PCR, including alcohol dehydrogenase 1 (*Adh1*), a proven direct target of GR (Edenberg and Brown, 1992), glycine N-methyltransferase (*Gnmt*) and hepatic lipase C (*Lipc*) (Fig. 8C). We observed significant differences in the expression of these genes at ZT8 in *Rev-erbα^−/−^* compared to wild-type livers. Interestingly, we identified *Insig2a* and *IκBα* (*Nfkbia*) in the 50 genes representing the overlap of REV-ERBα- and GR-dependent gene expression (Fig. 8B). We verified the difference in gene expression of the bona fide REV-ERBα target gene *Insig2a* (Le Martelot et al., 2009) and the GR target gene *IκBα* (*Nfkbia*) (Deroo and Archer, 2001). Both genes were upregulated in *Rev-erbα^−/−^* livers (Fig. 8D).

In order to test whether the verified genes in Fig. 8C,D are directly regulated by GR, we performed chromatin immunoprecipitation (ChIP) experiments. As control, we used the *Per1* promoter, which contains a GR response element (GRE) (Reddy et al., 2009). Probes unrelated to the GRE for the *Per1* promoter indicated the background signal for the ChIP experiments (Fig. 8E, top middle panel). Using specific probes flanking the GRE we observed a binding of GR to the GRE that was dependent on the time of day element in the *Per1* promoter in wild-type, with low or no binding at ZT8 and binding at ZT20, but constant chromatin binding in liver derived from *Rev-erbα^−/−^* mice (Fig. 8E, top left panel). Similar GR binding was observed for *Insig2*, *Adh1*, *Gnmt* and *Lipc* (Fig. 8E). These results indicate that these genes are regulated at least in part by GR, suggesting that the increase in gene expression observed in *Rev-erbα^−/−^* mice might stem from GR-related transcriptional mechanisms and not necessarily from lack of REV-ERBα-mediated transcriptional repression.

Taken together, these experiments support our hypothesis that REV-ERBα and GR influence each other, affecting expression of their respective target genes.

## DISCUSSION

### REV-ERBα and GR influence each other

GR (*Nr3c1*) and REV-ERBα (*Nr1d1*) both are nuclear receptors that modulate similar physiological pathways, including metabolism and inflammation (Tronche et al., 1998; Buckingham, 2006; Everett and Lazar, 2014). We previously observed that *Nr3c1* mRNA expression is downregulated in the suprachiasmatic nuclei (SCN) of *Rev-erbα^−/−^* mice compared to wild-type animals (Schnell et al., 2014). This prompted us to test a potential relationship between REV-ERBα and GR. Interestingly, we found that, in the liver, GR protein in *Rev-erbα^−/−^* mice is upregulated compared to in wild type (Fig. 1A,B). However, at the mRNA level, no differences in *Nr3c1* expression between the two genotypes were observed (Fig. 1C). These findings indicate that GR in the liver is not regulated in the same manner as in the SCN and that regulation in the liver most likely occurs at the post-transcriptional level (Fig. 1F).

In NIH 3T3 cells, we observed an inverse relationship between REV-ERBα and GR. The more REV-ERBα was expressed in the cells the less GR was detected (Fig. 2A). Given that GR interacts with heat-shock protein 90 (HSP90) before hormone-dependent activation (Kirschke et al., 2014), we wondered whether REV-ERBα would affect binding of GR to HSP90. We found that REV-ERBα interacts with HSP90 in a dose-dependent manner (Fig. 2B) influencing GR binding to HSP90 in an inverse fashion. This was confirmed in liver tissue from *Rev-erbα^−/−^* mice (Fig. 2C). The observation that the half-life of GR protein was shortened in presence of the REV-ERBα expression plasmid corroborated the above findings (Fig. 2D). Interestingly, however, REV-ERBα also affected the half-life of HSP90 (Fig. 2D). Hence, stability of GR could be mediated either directly or indirectly through modulation of stability of HSP90 upon changes in the amount of REV-ERBα. Taken together, we identified an inverse correlation between REV-ERBα and GR, which is most likely related to binding of these two nuclear receptors to HSP90.

Nuclear receptors regulate transcription and, hence, their activity depends on their localization to the nucleus. Using immunofluorescence and confocal microscopy we observed time-dependent subcellular distribution changes in GR and REV-ERBα in liver tissue (Figs 3, 4 and 5). In wild-type liver, GR was localized predominantly to the cytoplasm at ZT8 whereas it was nuclear at ZT20 (Fig. 4). In contrast, REV-ERBα was distributed between cytoplasm and nucleus in an inverse manner (Fig. 3). In the absence of REV-ERBα the time-dependent subcellular distribution changes in GR was not observed anymore (Fig. 4), indicating that REV-ERBα might play a role in this process. We do not know what the role of REV-ERBβ is, but it is probably not strong, because it cannot compensate for the loss of REV-ERBα. Our interpretation of the data is that REV-ERBα influences the subcellular distribution of GR through interaction with HSP90. However, we cannot exclude that REV-ERBα influences the machinery for translocation of GR into the nucleus. Corticosterone seems not to be a factor, though, because corticosterone levels in *Rev-erbα^−/−^* mice are not different compared to in wild type (Fig. 5B), as observed previously (Delezie et al., 2012).

### Sequential interaction of REV-ERBα and GR at different sites on HSP90

In order to investigate the temporal profile of REV-ERBα and GR interaction with HSP90 we analyzed total liver extracts collected at 4-h intervals from ZT0–ZT20 of wild-type and *Rev-erbα^−/−^* mice (Fig. 6A). We observed a sequential interaction of these two nuclear receptors with HSP90 between ZT0 and ZT12 with an overlap at ZT4. This indicates that the GR interaction with HSP90 disappeared upon the emergence of REV-ERBα interaction. Because the binding site of the two nuclear receptors to HSP90 appears not to be identical (Fig. 6B), a direct competition for the same binding site on HSP90 can be excluded. Possibly structural changes of HSP90 due to REV-ERBα binding might reduce the affinity of HSP90 for GR and release it. This possibility is supported by the observation that REV-ERBα protein levels start to increase at ZT4 with a maximum at ZT8 in liver tissue (Preitner et al., 2002), which coincides with the observed binding to HSP90 (Fig. 6A). Because most of the REV-ERBα protein appears to be nuclear at ZT8 (Fig. 3), the cytoplasmic HSP90-bound form of this nuclear receptor is most likely a differently modified or a truncated version. Evidence for a modified form of REV-ERBα can be seen in our overexpression experiment in Fig. 2D, where a double band of REV-ERBα protein was detected (Fig. 2D, 0 h after CHX treatment). It is known that REV-ERBα is phosphorylated and stabilized by GSK3β phosphorylation at serine residues 55 and 59 (Yin et al., 2006), and there might even be more modifications. Hence, post-translational modifications are important for the stability and might be important for the transport of REV-ERBα from the cytoplasm to the nucleus. Variants of REV-ERBα stemming from such processes are most likely dependent on the time of day, because at the time when REV-ERBα is predominantly cytoplasmic (ZT20) (Fig. 3) no interaction with HSP90 was observed (Fig. 6A).

Different forms of GR (splicing variants, translational initiation variants or post-translational modification variants; reviewed in Vandevyver et al., 2014) might also contribute to the inverse subcellular distribution observed in comparison to REV-ERBα. Evidence for this can be seen in Fig. 2C, where the input shows a double band for GR, which disappears after immunoprecipitation with HSP90 (Fig. 2C). This might affect REV-ERBα and its interaction with HSP90 and its subsequent subcellular localization. Taken together, it appears that the inverse subcellular localization we observed between GR and REV-ERBα is mechanistically very complex and not a simple competition between two nuclear receptors for binding to HSP90. Given that HSP90 is a very abundant protein in the cytoplasm, interaction with it is probably regulated by post-translational modification of its binding partners.

### REV-ERBα, GR and inflammation

Diurnal variation of inflammatory function has been described (Castanon-Cervantes et al., 2010), but molecular mechanisms mediating its gating are unknown. Disruption of the circadian clock by deleting *Bmal1* specifically in macrophages has been shown to remove all temporal gating of endotoxin-induced responses (Gibbs et al., 2012). This loss of circadian gating was coincident with suppressed *Rev-erbα* expression. Given that REV-ERBα has been reported to modulate expression of NF-κB-responsive genes in humans (Migita et al., 2004) and GR exerts anti-inflammatory action in part by antagonizing NF-κB through upregulation of IκBα (Scheinman et al., 1995; Almawi and Melemedjian, 2002), whose promoter contains GR-binding sites (Lim et al., 2015), we treated primary cultures of hepatocytes from control and *Rev-erbα^−/−^* mice with ethanol to activate this pathway (Fig. 7, Fig. S1). We found that this signaling pathway is inhibited in hepatocytes of *Rev-erbα^−/−^* mice, but knocking down GR or IκBα led to an almost normal induction of this signaling pathway as manifested by the induction of TNFα. Hence, REV-ERBα appears to be a link between the clock and immune function, most likely through its potential to interfere with the interaction of GR with HSP90, thereby gating nuclear entry of GR to ZT20 by an unknown and probably indirect mechanism (Fig. 4). This provides a rational for the observation that in mice with a bronchiole-specific deletion of *Bmal1*, circadian binding of GR at the *Cxcl5* locus is disrupted (Gibbs et al., 2014). In these animals REV-ERBα expression is strongly reduced, and the presence of GR in the nucleus would not be gated anymore. Accordingly, *Cxcl5* would not be rhythmically, but rather constantly expressed, which has been observed in lung tissue of these animals (Gibbs et al., 2014). Taken together, this indicates that gating of GR to the nucleus involving REV-ERBα most likely does not only occur in liver but in other tissues such as the lung as well.

### REV-ERBα, GR and metabolism

Metabolism is affected in both *Rev-erbα* and GR liver-specific knockout mice (Opherk et al., 2004; Bugge et al., 2012; Cho et al., 2012). Mice lacking GR in the liver display faster phase-resetting of the liver clock upon restricted feeding (Le Minh et al., 2001), and *Rev-erbα^−/−^* mice exhibit a reduction in food anticipatory behavior (Delezie et al., 2012). This illustrates that metabolism is modulated by these two nuclear receptors.

Our data support this view. For example, alcohol dehydrogenase 1 (*Adh1*) expression is altered in the liver of *Rev-erbα^−/−^* mice (Fig. 8C) and is regulated by GR, as evidenced by ChIP (Fig. 8E), illustrating the potential consequence of lack of REV-ERBα on GR expression at ZT8. Similar effects were observed for glycine N-methyltransferase (*Gnmt*) and hepatic lipase C (*Lipc*), enzymes involved in liver detoxification and lipid metabolism, respectively. Although these genes appear to be regulated by GR (Fig. 8E), the pathways they are involved in are also altered in *Rev-erbα^−/−^* mice (Le Martelot et al., 2009; Bugge et al., 2012; Cho et al., 2012). Most interestingly, Insig2 an oxysterol sensor, that sequesters sterol-regulatory-element-binding proteins to the endoplasmic reticulum and thereby indirectly modulates fatty acid and cholestorol metabolism, has been identified as a bona fide REV-ERBα target (Le Martelot et al., 2009). Our data confirm the change of *Insig2a* gene expression in *Rev-erbα^−/−^* mice (Fig. 8D) with the twist that this gene can be regulated by GR, as evidenced by ChIP (Fig. 8E). These results suggest a close relationship between REV-ERBα- and GR-mediated transcriptional regulation.

In summary, we show in this study that REV-ERBα influences the subcellular distribution of the GR protein by an unknown mechanism. As a consequence these two nuclear receptors influence the transcriptional potential of each other leading to a temporal segregation of GR target gene expression. This illustrates the complex cross-regulation between nuclear receptors, providing an explanation for off-target effects of drugs modulating specific nuclear receptor functions.

## MATERIALS AND METHODS

### Animals

Animal care and handling was performed in accordance with the guidelines of the Schweizer Tierschutzgesetz (TSchG, SR455) and the declaration of Helsinki authorized by the Office Veterinaire Cantonal de Fribourg. *Rev-erba-*knockout mice (Preitner et al., 2002, 129/C57BL6 mixed background) were obtained from heterozygous breeding pairs originally provided by Prof. Ueli Schibler (Department of Molecular Biology, University of Geneva, Geneva). Two- to four-month-old male mice were used for experiments and wild-type mice served as controls. Animals were kept under 12 h light and 12 h dark conditions with food and water *ad libitum*.

### Cell culture

NIH3T3 mouse fibroblast cells (ATCC^R^CRL-1658™) were maintained in Dulbecco's modified Eagle's medium (DMEM), containing 10% fetal calf serum (FCS) and 100 U/ml penicillin-streptomycin at 37°C in a humidified atmosphere containing 5% CO_2_. NIH3T3 cells were treated with 100 µM cycloheximide after transfection containing either empty vector or PSCT1-Rev-erbα. Cells were harvested 0, 2, 4 and 6 h after treatment.

### Quantitative real-time PCR

Total RNA was extracted from snap-frozen liver tissue using RNA-Bee (AMS Biotechnology), according to the manufacturer's instructions. Liver RNA was precipitated in 4 M LiCl to remove glycogen, and was purified further by phenol:chloroform extraction and ethanol precipitation. Single-stranded DNA (ssDNA) complementary to the RNA starting from hybridized random hexamer primers was synthesized with SuperScript II (Life Technology Corporation) according to the manufacturer's instructions. SYBR green fluorescence-based qRT-PCR was performed for RNA quantification (KAPA SYBR FAST Universal, KAPA Biosystems, RotorGene 6000, Corbett Research). All RNA samples were normalized to *Gapdh*. Primers are listed in Table S2.

### Luciferase reporter assays and transfection

A 1756-bp fragment of the mouse Nr3c1 promoter region (−1750 to +6 bp from the transcription start site containing RORE elements) was cloned into the pGL3 basic vector (Promega) using the following primers: 5′-CGGTACCTGAAGTCGTGTCCTTAC-3′ (sense primer) and 5′-CCTCGAGGTCTCCAAGAGAAACAC-3′ (anti-sense primer). Transfection and luciferase reporter assays were performed with NIH3T3 cells according to Schnell et al. (2014). The *Bmal1* promoter region cloned into pGL3 were used as positive controls.

### Western blotting analysis

Protein of cultured cells and liver tissue was extracted using RIPA buffer (50 mM Tris-HCl pH 7.4, 150 mM NaCl, 1 mM EDTA, 0.1% SDS, 1% Triton X-100, 0.5% sodium deoxycholate containing protease and phosphatase inhibitors). Protein samples (40 μg) were subjected to electrophoresis on 10% SDS-polyacrylamide gels and transferred to a nitrocellulose (Amersham Protran Supported 0.45 NC, GE healthcare). After blocking with 0.5% dry milk in PBS-Tween 0.1%, the membranes were probed with antibodies against GR (1:500, Santa Cruz Biotechnology, sc-1004), HSP90α/β (F-8) (1:1000, Santa Cruz Biotechnology, sc-13119), REV-ERBα (1:200, Santa Cruz Biotechnology, sc-100910), BMAL1 (1:500, Ripperger and Schibler, 2006), NF-κB p65 (1:200, Santa Cruz Biotechnology, sc-109), IκBα (1:200, Santa Cruz Biotechnology, sc-371), phosphorylated (p-)IκBα (1:200, Santa Cruz Biotechnology, sc-8404), tubulin (1:1000, Abcam, ab 15246) or LAMINB1 (1:500, Santa Cruz Biotechnology, sc-30264) overnight at 4°C. Anti-rabbit-IgG, mouse-IgG and goat-IgG antibody conjugated to horseradish peroxidase (HPR) was used as a secondary antibody. Detection of the immune complexes was performed using Western Bright Quantum system (Advansta) and quantification was done with the Quantity One analysis software (BioRad).

### Pulldown assay with HSP90 fragments

GST-fused recombinant HSP90 proteins were expressed in an *E. coli* BL21 strain [plasmids: GST-Hsp90 N(9-236), GST-Hsp90M(272-617), and GST-Hsp90 C(626-732), Addgene 22481, 22482 and 22483, respectively]. Proteins were purified to homogeneity with glutathione–agarose beads for 2 h at 4°C. The beads were then incubated overnight at 4°C with purified GR and REV-ERBα both containing a His-tag (GR-LBD: pET15Avi6HT-Nr3c1 DNASU plasmid no. HsCD00651869; Rev-erbα: pET15bxRev-erbα). Subsequently, elution with 10 mM reduced glutahione took place for 15 min at room temperature. Elution was stopped by adding Laemmli buffer and samples were loaded onto the gel after 10 min at 95°C.

### Preparation of cytoplasm and nuclear extracts from primary cultured hepatocytes

The isolated hepatocytes nuclear extracts were washed with PBS and then were homogenized in ice-cold homogenization buffer (10 mM HEPES pH 7.9, 1.5 mM MgCl_2_, 10 mM KCl, 1 mM DTT, 0.1 mM EDTA and 0.1% NP40) containing protease inhibitor (1 μg/ml). The homogenates were incubated on ice for 30 min and nuclei were collected by centrifugation at 800 ***g*** for 5 min at 4°C. Nuclei were resuspended in extraction buffer (20 mM HEPES pH 7.9, 10% glycerol, 400 mM NaCl, 1.5 mM MgCl_2_, 0.1 mM EDTA, 1 mM DTT, and 0.1% NP40) containing protease inhibitor. After incubation on ice for 1 h, cell debris and DNA was removed by centrifugation at 21,000 ***g*** for 10 min at 4°C. Extracts were aliquoted and stored at −80°C.

### Co-immunoprecipitation

For co-immunoprecipitation experiments with cultured cells and mouse whole-liver extracts, 400 μg of whole extracts were incubated with rotation overnight at 4°C with the indicated antibody and captured with protein-A–agarose beads (Roche Applied Science) for 4 h at 4°C. The beads were washed four times with RIPA buffer. After washes, the samples were resuspended in 4× SDS protein sample buffer (40% glycerol, 240 mM Tris-HCl pH 6.8, 8% SDS, 0.04% Bromophenol Blue, 5% β-mercaptoethanol) and subjected to SDS-PAGE.

### Mouse hepatocyte isolation

Hepatocytes were isolated from livers of *Rev-erbα*^−/−^ and wild-type mice using collagenase perfusion with modifications. The inferior vena cava was cannulated and the liver was first perfused *in situ* with a Hanks' buffer salt solution (HBSS), pH 7.4 (4 ml/min, 37°C for 10 min), followed by perfusion with HBSS containing 1 mM Ca^2+^ and Mg^2+^ and 0.01% collagenase type 1 (Thermo Fisher Sientific), pH 7.4, for 10 min. The liver was removed and then gently minced in HBSS containing 1 mM Ca^2+^ and Mg^2+^ and 0.01% collagenase type 1, pH 7.4. The liver cell suspension was then filtered with Falcon cell strainers (70 mm; Becton Dickinson, Bedford, MA) and centrifuged at 50 ***g*** for 10 min. Cells were plated on a collagen-I-coated plate (Thermo Fisher Sientific), (6×105 cells/well) in DMEM containing 10% FCS and 100 U/ml penicillin-streptomycin at 37°C in a humidified atmosphere containing 5% CO_2_. After an initial 5-h attachment period, cultures were washed with PBS and then fresh culture medium.

### Immunohistochemistry

Animals used for the immunohistochemistry were killed at ZT8 and ZT20. Brains were perfused with 0.9% NaCl and 4% paraformaldehyde (PFA). Perfused brains were cryoprotected by 30% sucrose solution, and sectioned (15 μm) using a cryostat. Sections chosen for staining were washed three times with 1× TBS and 2× SSC (pH 7, 0.3 M NaCl/0.03 M tri-Na-citrate). Antigen retrieval was performed with 50% formamide and 2× SSC by heating to 65°C for 60 min. Later, sections were washed in 2× SSC and in 1× TBS pH 7.5 (0.1 M Tris-HCl, 0.15 M NaCl), before blocking them for 2 h in 10% normal donkey serum sterile (NDS, ab138579, abcam, UK), 0.1% Triton X-100 and 1× TBS at room temperature. After the blocking, primary anti-Rev-Erbα antibody (ab174309, abcam, 1:100), anti-GR (Santa Cruz Biotechnology, sc-1004, 1:50) and anti-HSP90 antibody (Santa Cruz Biotechnology, sc-13119, 1:50), diluted in 1% normal donkey serum (NDS), 0.1% Triton X-100 and 1× TBS were added to the sections and incubated 48 h at 4°C. Sections were washed with in 1× TBS and incubated with the appropriate fluorescent secondary antibodies diluted 1:500 in 1% NDS, 0.1% Triton X-100 and 1× TBS for 3 h at room temperature [Alexa Fluor^®^ 488-AffiniPure donkey anti-rabbit IgG (H+L), 711-545-152, and Alexa Fluor^®^ 647-AffiniPure donkey anti-mouse IgG (H+L), 715-605-150; Jackson Immuno Research]. After washing with 1× TBS, nuclei were counterstained with DAPI (300 nM) for 15 min. Finally, the tissue sections were washed again twice in 1× TBS and mounted on glass microscope slides. Fluorescence images were taken by using a confocal microscope (Leica TCS SP5), and images were taken with objective 20× a resolution of 1024×1024, scan speed 200 Hz and *z*-stack of 1.5 μm through whole section with frame average of 3. Images were processed with LAS AS software from LEICA.

### Preparation of cytoplasm and nuclear extracts from liver

Small piece of frozen liver was homogenized in ice-cold homogenization buffer (100 mM Tris-HCl, 10 mM DTT). The homogenates were incubated on ice for 10 min and centrifuged at 2900 ***g*** for 5 min at 4°C. Pellets were resuspended in 200 μl complete cytoplasm lysis buffer (10 mM EDTA, 10 mM Hepes, 5 mM NaF, 0.2 mM Na Vanadate, 2 mM DTT, and 0.2% Triton X-100) containing protease inhibitor (Roche) and centrifuged at 2900 ***g*** for 5 min at 4°C. Supernatant were collected as cytoplasm extracts. After three washes with complete cytoplasm lysis buffer, nuclei were resuspended in 100 μl NDB buffer (20% glycerol, 20 mM Hepes, 0.2 mM EDTA, 10 mM NaF, 0.4 mM Na Vanadate, 2 mM DTT and 100 mM KCl) containing protease inhibitor and 100 μl NUN buffer (2 M urea, 600 mM NaCl, 2% NP-40, and 50 mM Hepes) were added. After incubation on ice for 30 min, cell debris and DNA was removed by centrifugation at 15,700 ***g*** for 30 min at 4°C. Supernatant was collected as for nuclear extracts.

### Plasma corticosterone measurement

Under gaseous isoflourane anesthesia, blood samples were collected from the tail vein at different time points (ZT8 and ZT20). Plasma was recovered by centrifuging samples at 1500 ***g*** for 10 min in an Eppendorf centrifuge. Plasma corticosterone levels were measured with the Corticosterone EIA kit (ADI-900-097, Enzo Life Sciences) according to the manufacturer's instructions.

### Knockdown of GR and IκBα, ethanol treatment and measurement of TNF

At 24 h after seeding cells, shRNA lentiviral particles (GR; sc-35506-V, IκBα; sc-29361-V, Santa Cruz Biotechnology) were used for knocking down GR and IκBα according to the manufacturer's instructions. Knockdown efficiency was assessed at 48 h post transduction by western blotting. Scrambled shRNA lentiviral particles (sc-108080, Santa Cruz Biotechnology) was used as a negative control. At 24 h after transduction, dexamethasone was added to a final concentration of 1 μM. After 24 h, the culture medium was changed for one with or without 100 mM ethanol. After 24 h, the medium was collected and the levels of TNF were measured with the TNF alpha ELISA Kit (Thermo Fischer Scientific) according to the manufacturer's instructions. Cells were washed twice with phosphate buffer, scraped and subjected to protein extraction.

### RNA extraction and library construction

Liver samples were immediately flash frozen in liquid N_2_ and stored at −80°C. RNA was extracted using NucleoSpin RNA (Machery-Nagel, Düren, Germany) according to the instructions of the manufacturer. The quality of the RNA samples was analyzed with a spectrophotometer, agarose gel electrophoresis and reverse-transcription-PCR. Library construction starting from the poly(A)-tail and multiplexing was performed according to the instructions of the manufacturer (Illumina). The samples were organized as follows: three replicas (1-WT, 2-WT, 3-WT) correspond to genotype WT at ZT8; three replicas (4-Rev, 5-Rev, 6-Rev) correspond to genotype *Rev-erbα*^−/−^ at ZT8; three replicas (7-WT, 8-WT, 9-WT) correspond to genotype WT at ZT20; three replicas (10-Rev, 11-Rev, 12-Rev) correspond to genotype *Rev-erbα*^−/−^ at ZT20.

For the experiment, complementary DNA (cDNA) libraries were barcoded using Illumina primers and loaded onto one lane of an IlluminaHS2000 machine. cDNA libraries were diluted and loaded onto each lane. The samples were sequenced for a maximum sequencing length of 75 bp.

### Analysis of RNA-Seq data sets

Sequences were aligned to the mouse genome (UCSC version mm10 database). Numbers of the sequences obtained for each library can be found in Table S3. Sequences (fastq format) were mapped with Tophat (Trapnell et al., 2009), uniquely mapped sequences from the output files (bam format) were then used for further analysis, the percentage of the mapping obtained for each sample can be found in Table S3. For all files, the reads were counted with HTSeq-count using the following criteria:

samtools view sample.bam | htseq-count -m union -a 10 -s no -i gene_name Mus_Musculus.gtf>sample_counts.txt.

Tests for differential expression between the samples were performed in R software (R Core Team, 2014 http://www.R-project.org/) using the DESeq2 package (Version 1.6.3) (Love et al., 2014). A threshold on the corrected *P* value was used to call for differentially expressed genes (*P*.adjust<0.05).

The RNA-seq data have been deposited in NCBI Gene Expression Omnibus database under accession code GSE79087.

### Pathway analysis

The analysis was performed using official web-based set of tools for searching and browsing the Gene Ontology database AmiGO 2 (Carbon et al., 2009) (http://amigo.geneontology.org/amigo). Analysis of GO data was done using term enrichment analysis, finding significant shared GO terms or parents of those GO terms to help discover what input genes might be in common (Mi et al., 2013).

### Chromatin immunoprecipitation

ChIP was performed as described previously (Ripperger and Schibler, 2006) using an anti-GR antibody at a dilution of 1:25 (M-20, sc-1004, Santa Cruz Biotechnology). Binding sites for GR in the 5′-ends of target genes were derived from a published ChIP sequencing experiment (GSM1446065 and GSM1446066, Lim et al., 2015), comparing the binding of GR to mouse liver chromatin at two different time points (6 am versus 6 pm). The probes to detect the immunoprecipitated DNA fragments are listed in Table S4.

### Statistical analysis

Statistical evaluation of all experiments was performed using GraphPad Prism6 software. Depending on the type of data, either an unpaired *t*-test, or one- or two-way ANOVA with Bonferroni or Tukey's post-hoc test was performed. Values were considered significantly different are highlighted [*P*<0.05 (*), *P*<0.01 (**), or *P*<0.001 (***)].

## References

[JCS190959C1] Almawi W. Y. and Melemedjian O. K. (2002). Molecular mechanisms of glucocorticoid antiproliferative effects: antagonism of transcription factor activity by glucocorticoid receptor. *J. Leukoc. Biol.* 71, 9-15. 11781376

[JCS190959C2] Beier J. I. and McClain C. J. (2010). Mechanisms and cell signaling in alcoholic liver disease. *Biol. Chem.* 391, 1249-1264. 10.1515/bc.2010.137 20868231 PMC3755482

[JCS190959C3] Berg J. M. (1989). DNA binding specificity of steroid receptors. *Cell* 57, 1065-1068. 10.1016/0092-8674(89)90042-1 2661016

[JCS190959C4] Buckingham J. C. (2006). Glucocorticoids: exemplars of multi-tasking. *Br. J. Pharmacol.* 147 Suppl. 1, S258-S268. 10.1038/sj.bjp.0706456 16402112 PMC1760726

[JCS190959C5] Bugge A., Feng D., Everett L. J., Briggs E. R., Mullican S. E., Wang F., Jager J. and Lazar M. A. (2012). Rev-erbalpha and Rev-erbbeta coordinately protect the circadian clock and normal metabolic function. *Genes Dev.* 26, 657-667. 10.1101/gad.186858.112 22474260 PMC3323877

[JCS190959C6] Buhr E. D. and Takahashi J. S. (2013). Molecular components of the Mammalian circadian clock. *Handb. Exp. Pharmacol.* 217, 3-27. 10.1007/978-3-642-25950-0_1 PMC3762864 23604473

[JCS190959C7] Carbon S., Ireland A., Mungall C. J., Shu S., Marshall B. and Lewis S., AmiGO hub web presence working group (2009). AmiGO: online access to ontology and annotation data. *Bioinformatics* 25, 288-289. 10.1093/bioinformatics/btn615 19033274 PMC2639003

[JCS190959C8] Castanon-Cervantes O., Wu M., Ehlen J. C., Paul K., Gamble K. L., Johnson R. L., Besing R. C., Menaker M., Gewirtz A. T. and Davidson A. J. (2010). Dysregulation of inflammatory responses by chronic circadian disruption. *J. Immunol.* 185, 5796-5805. 10.4049/jimmunol.1001026 20944004 PMC2974025

[JCS190959C10] Cho H., Zhao X., Hatori M., Yu R. T., Barish G. D., Lam M. T., Chong L.-W., DiTacchio L., Atkins A. R., Glass C. K. et al. (2012). Regulation of circadian behaviour and metabolism by REV-ERB-alpha and REV-ERB-beta. *Nature* 485, 123-127. 10.1038/nature11048 22460952 PMC3367514

[JCS190959C11] De Bosscher K., Vanden Berghe W. and Haegeman G. (2000). Mechanisms of anti-inflammatory action and of immunosuppression by glucocorticoids: negative interference of activated glucocorticoid receptor with transcription factors. *J. Neuroimmunol.* 109, 16-22. 10.1016/S0165-5728(00)00297-6 10969176

[JCS190959C12] De Bosscher K., Vanden Berghe W. and Haegeman G. (2003). The interplay between the glucocorticoid receptor and nuclear factor-kappaB or activator protein-1: molecular mechanisms for gene repression. *Endocr. Rev.* 24, 488-522. 10.1210/er.2002-0006 12920152

[JCS190959C13] Delezie J., Dumont S., Dardente H., Oudart H., Grechez-Cassiau A., Klosen P., Teboul M., Delaunay F., Pevet P. and Challet E. (2012). The nuclear receptor REV-ERBalpha is required for the daily balance of carbohydrate and lipid metabolism. *FASEB J.* 26, 3321-3335. 10.1096/fj.12-208751 22562834

[JCS190959C14] Deroo B. J. and Archer T. K. (2001). Glucocorticoid receptor activation of the Ikappa Balpha promoter within chromatin. *Mol. Biol. Cell* 12, 3365-3374. 10.1091/mbc.12.11.3365 11694573 PMC60261

[JCS190959C15] Downes M., Carozzi A. J. and Muscat G. E. (1995). Constitutive expression of the orphan receptor, Rev-erbA alpha, inhibits muscle differentiation and abrogates the expression of the myoD gene family. *Mol. Endocrinol.* 9, 1666-1678 10.1210/mend.9.12.8614403 8614403

[JCS190959C16] Edenberg H. J. and Brown C. J. (1992). Regulation of human alcohol dehydrogenase genes. *Pharmacogenetics* 2, 185-196. 10.1097/00008571-199210000-00001 1339084

[JCS190959C17] Evans R. M. (1988). The steroid and thyroid hormone receptor superfamily. *Science* 240, 889-895. 10.1126/science.3283939 3283939 PMC6159881

[JCS190959C18] Everett L. J. and Lazar M. A. (2014). Nuclear receptor Rev-erbalpha: up, down, and all around. *Trends Endocrinol. Metab.* 25, 586-592. 10.1016/j.tem.2014.06.011 25066191 PMC4252361

[JCS190959C19] Fan C., Li Q., Zhang Y., Liu X., Luo M., Abbott D., Zhou W. and Engelhardt J. F. (2004). IkappaBalpha and IkappaBbeta possess injury context-specific functions that uniquely influence hepatic NF-kappaB induction and inflammation. *J. Clin. Invest.* 113, 746-755. 10.1172/JCI17337 14991073 PMC351311

[JCS190959C20] Gao B., Seki E., Brenner D. A., Friedman S., Cohen J. I., Nagy L., Szabo G. and Zakhari S. (2011). Innate immunity in alcoholic liver disease. *Am. J. Physiol. Gastrointest. Liver Physiol.* 300, G516-G525. 10.1152/ajpgi.00537.2010 21252049 PMC3774265

[JCS190959C21] Gibbs J. E., Blaikley J., Beesley S., Matthews L., Simpson K. D., Boyce S. H., Farrow S. N., Else K. J., Singh D., Ray D. W. et al. (2012). The nuclear receptor REV-ERBalpha mediates circadian regulation of innate immunity through selective regulation of inflammatory cytokines. *Proc. Natl. Acad. Sci. USA* 109, 582-587. 10.1073/pnas.1106750109 22184247 PMC3258648

[JCS190959C22] Gibbs J., Ince L., Matthews L., Mei J., Bell T., Yang N., Saer B., Begley N., Poolman T., Pariollaud M. et al. (2014). An epithelial circadian clock controls pulmonary inflammation and glucocorticoid action. *Nat. Med.* 20, 919-926. 10.1038/nm.3599 25064128 PMC4268501

[JCS190959C23] Kirschke E., Goswami D., Southworth D., Griffin P. R. and Agard D. A. (2014). Glucocorticoid receptor function regulated by coordinated action of the Hsp90 and Hsp70 chaperone cycles. *Cell* 157, 1685-1697. 10.1016/j.cell.2014.04.038 24949977 PMC4087167

[JCS190959C24] Kumar N., Solt L. A., Wang Y., Rogers P. M., Bhattacharyya G., Kamenecka T. M., Stayrook K. R., Crumbley C., Floyd Z. E., Gimble J. M. et al. (2010). Regulation of adipogenesis by natural and synthetic REV-ERB ligands. *Endocrinology* 151, 3015-3025. 10.1210/en.2009-0800 20427485 PMC2903944

[JCS190959C25] Laitinen S., Fontaine C., Fruchart J. C. and Staels B. (2015). The role of the orphan nuclear receptor Rev-erb alpha in adipocyte differentiation and function. *Biochimie* 1, 21-25. 10.1016/j.biochi.2004.12.006 15733732

[JCS190959C26] Le Martelot G., Claudel T., Gatfield D., Schaad O., Kornmann B., Lo Sasso G., Moschetta A. and Schibler U. (2009). REV-ERBalpha participates in circadian SREBP signaling and bile acid homeostasis. *PLoS Biol.* 7, e1000181 10.1371/journal.pbio.1000181 19721697 PMC2726950

[JCS190959C27] Le Minh N., Damiola F., Tronche F., Schutz G. and Schibler U. (2001). Glucocorticoid hormones inhibit food-induced phase-shifting of peripheral circadian oscillators. *EMBO J.* 20, 7128-7136. 10.1093/emboj/20.24.7128 11742989 PMC125339

[JCS190959C28] Lim H.-W., Uhlenhaut N. H., Rauch A., Weiner J., Hübner S., Hübner N., Won K.-J., Lazar M. A., Tuckermann J. and Steger D. J. (2015). Genomic redistribution of GR monomers and dimers mediates transcriptional response to exogenous glucocorticoid in vivo. *Genome Res.* 25, 836-844. 10.1101/gr.188581.114 25957148 PMC4448680

[JCS190959C29] Love M. I., Huber W. and Anders S. (2014). Moderated estimation of fold change and dispersion for RNA-seq data with DESeq2. *Genome Biol.* 15, 550 10.1186/s13059-014-0550-8 25516281 PMC4302049

[JCS190959C30] Mi H., Murunganujan A., Casagrande J. T. and Thomas P. D. (2013). Large-scale gene function analysis with the PANTHER classification system. *Nat. Protoc.* 8, 1551-1566. 10.1038/nprot.2013.092 23868073 PMC6519453

[JCS190959C31] Migita H., Morser J. and Kawai K. (2004). Rev-erbalpha upregulates NF-kappaB-responsive genes in vascular smooth muscle cells. *FEBS Lett.* 561, 69-74. 10.1016/S0014-5793(04)00118-8 15013753

[JCS190959C32] Opherk C., Tronche F., Kellendonk C., Kohlmuller D., Schulze A., Schmid W. and Schutz G. (2004). Inactivation of the glucocorticoid receptor in hepatocytes leads to fasting hypoglycemia and ameliorates hyperglycemia in streptozotocin-induced diabetes mellitus. *Mol. Endocrinol.* 18, 1346-1353. 10.1210/me.2003-0283 15031319

[JCS190959C33] Panda S., Hogenesch J. B. and Kay S. A. (2002). Circadian rhythms from flies to human. *Nature* 417, 329-335. 10.1038/417329a 12015613

[JCS190959C34] Phuc Le P., Friedman J. R., Schug J., Brestelli J. E., Parker J. B., Bochkis I. M. and Kaestner K. H. (2005). Glucocorticoid receptor-dependent gene regulatory networks. *PLoS Genet.* 1, e16 10.1371/journal.pgen.0010016 16110340 PMC1186734

[JCS190959C35] Preitner N., Damiola F., Lopez-Molina L., Zakany J., Duboule D., Albrecht U. and Schibler U. (2002). The orphan nuclear receptor REV-ERBalpha controls circadian transcription within the positive limb of the mammalian circadian oscillator. *Cell* 110, 251-260. 10.1016/S0092-8674(02)00825-5 12150932

[JCS190959C36] Reddy T. E., Pauli F., Sprouse R. O., Neff N. F., Newberry K. M., Garabedian M. J. and Myers R. M. (2009). Genomic determination of the glucocorticoid response reveals unexpected mechanisms of gene regulation. *Genome Res.* 19, 2163-2171. 10.1101/gr.097022.109 19801529 PMC2792167

[JCS190959C37] Reppert S. M. and Weaver D. R. (2001). Molecular analysis of mammalian circadian rhythms. *Annu. Rev. Physiol.* 63, 647-676. 10.1146/annurev.physiol.63.1.647 11181971

[JCS190959C38] Reppert S. M. and Weaver D. R. (2002). Coordination of circadian timing in mammals. *Nature* 418, 935-941. 10.1038/nature00965 12198538

[JCS190959C39] Ripperger J. A. and Schibler U. (2006). Rhythmic CLOCK-BMAL1 binding to multiple E-box motifs drives circadian Dbp transcription and chromatin transitions. *Nat. Genet.* 38, 369-374. 10.1038/ng1738 16474407

[JCS190959C40] Scheinman R. I., Cogswell P. C., Lofquist A. K. and Baldwin A. S. Jr (1995). Role of transcriptional activation of I kappa B alpha in mediation of immunosuppression by glucocorticoids. *Science* 270, 283-286. 10.1126/science.270.5234.283 7569975

[JCS190959C41] Schnell A., Chappuis S., Schmutz I., Brai E., Ripperger J. A., Schaad O., Welzl H., Descombes P., Alberi L. and Albrecht U. (2014). The nuclear receptor REV-ERBalpha regulates Fabp7 and modulates adult hippocampal neurogenesis. *PLoS ONE* 9, e99883 10.1371/journal.pone.0099883 24932636 PMC4059695

[JCS190959C42] Siriani D., Mitsiou D. J. and Alexis M. N. (2005). Heat-induced degradation of overexpressed glucocorticoid receptor Separate protective roles of hsp90 and hsp70. *J. Steroid. Biochem. Mol. Biol.* 94, 93-101. 10.1016/j.jsbmb.2005.01.013 15862954

[JCS190959C43] Sultana R., Theodoraki M. A. and Caplan A. J. (2013). Specificity in the actions of the UBR1 ubiquitin ligase in the degradation of nuclear receptors. *FEBS Open Bio.* 3, 394-397. 10.1016/j.fob.2013.09.003 PMC3821023 24251101

[JCS190959C44] Trapnell C., Pachter L. and Salzberg S. L. (2009). TopHat: discovering splice junctions with RNA-Seq. *Bioinformatics* 25, 1105-1111. 10.1093/bioinformatics/btp120 19289445 PMC2672628

[JCS190959C45] Tronche F., Kellendonk C., Reichardt H. M. and Schutz G. (1998). Genetic dissection of glucocorticoid receptor function in mice. *Curr. Opin. Genet. Dev.* 8, 532-538. 10.1016/S0959-437X(98)80007-5 9794823

[JCS190959C46] Vandevyver S., Dejager L. and Libert C. (2014). Comprehensive overview of the structure and regulation of the glucocorticoid receptor. *Endocr. Rev.* 35, 671-693. 10.1210/er.2014-1010 24937701

[JCS190959C47] Wang F., Yang J.-L., Yu K.-K., Xu M., Xu Y.-Z., Chen L., Lu Y.-M., Fang H.-S., Wang X.-Y., Hu Z.-Q. et al. (2015). Activation of the NF-kappaB pathway as a mechanism of alcohol enhanced progression and metastasis of human hepatocellular carcinoma. *Mol. Cancer* 14, 10 10.1186/s12943-014-0274-0 25622857 PMC4320626

[JCS190959C48] Woldt E., Sebti Y., Solt L. A., Duhem C., Lancel S., Eeckhoute J., Hesselink M. K. C., Paquet C., Delhaye S., Shin Y. et al. (2013). Rev-erb-alpha modulates skeletal muscle oxidative capacity by regulating mitochondrial biogenesis and autophagy. *Nat. Med.* 19, 1039-1046. 10.1038/nm.3213 23852339 PMC3737409

[JCS190959C49] Yin L., Wang J., Klein P. S. and Lazar M. A. (2006). Nuclear receptor Rev-erbα is a critical lithium-sensitive component of the circadian clock. *Science* 311, 1002-1005. 10.1126/science.1121613 16484495

